# Epidemiology of Carbapenem-resistant Enterobacteriaceae in Egyptian intensive care units using National Healthcare–associated Infections Surveillance Data, 2011–2017

**DOI:** 10.1186/s13756-019-0639-7

**Published:** 2020-01-03

**Authors:** Sara Kotb, Meghan Lyman, Ghada Ismail, Mohammad Abd El Fattah, Samia A. Girgis, Ahmed Etman, Soad Hafez, Jehan El-Kholy, Maysaa El Sayed Zaki, Hebat-allah G. Rashed, Ghada M. Khalil, Omar Sayyouh, Maha Talaat

**Affiliations:** 1 Division of Global Health Protection, US Centers for Disease Control and Prevention, Cairo, Egypt; 2 Centers for Disease Control and Prevention, Atlanta, GA USA; 3 Supreme Council of Universities, Cairo, Egypt; 4 Ministry of Health and Population, Cairo, Egypt; 5 Ain Shams University Hospitals, Cairo, Egypt; 6 Alexandria University Hospitals, Alexandria, Egypt; 7 Cairo University Hospitals, Cairo, Egypt; 8 Mansoura University Hospitals, Mansoura, Egypt; 9 Assiut University Hospitals, Assiut, Egypt; 10 Zagazig University Hospital, Benha, Egypt

**Keywords:** Antimicrobial resistance, Carbapenem resistance Enterobacteriaceae, Healthcare-associated infections

## Abstract

**Objective:**

To describe the epidemiology of carbapenem-resistant Enterobacteriaceae (CRE) healthcare-associated infections (HAI) in Egyptian hospitals reporting to the national HAI surveillance system.

**Methods:**

Design**:** Descriptive analysis of CRE HAIs and retrospective observational cohort study using national HAI surveillance data**.** Setting: Egyptian hospitals participating in the HAI surveillance system. The patient population included patients admitted to the intensive care unit (ICU) in participating hospitals**.** Enterobacteriaceae HAI cases were *Klebsiella, Escherichia coli,* and *Enterobacter* isolates from blood, urine, wound or respiratory specimen collected on or after day 3 of ICU admission. CRE HAI cases were those resistant to at least one carbapenem. For CRE HAI cases reported during 2011–2017, a hospital-level and patient-level analysis were conducted using only the first CRE isolate by pathogen and specimen type for each patient. For facility, microbiology, and clinical characteristics, frequencies and means were calculated among CRE HAI cases and compared with carbapenem-susceptible Enterobacteriaceae HAI cases through univariate and multivariate logistic regression using STATA 13.

**Results:**

There were 1598 Enterobacteriaceae HAI cases, of which 871 (54.1%) were carbapenem resistant. The multivariate regression analysis demonstrated that carbapenem resistance was associated with specimen type, pathogen, location prior to admission, and length of ICU stay. Between 2011 and 2017, there was an increase in the proportion of Enterobacteriaceae HAI cases due to CRE (*p*-value = 0.003) and the incidence of CRE HAIs (*p*-value = 0.09).

**Conclusions:**

This analysis demonstrated a high and increasing burden of CRE in Egyptian hospitals, highlighting the importance of enhancing infection prevention and control (IPC) programs and antimicrobial stewardship activities and guiding the implementation of targeted IPC measures to contain CRE in Egyptian ICU’s .

## Background

Antimicrobial resistance (AMR) is being increasingly recognized as a global health security threat that requires integrated action across government sectors and society as a whole [1]. Carbapenem-resistant Enterobacteriaceae (CRE) are especially concerning pathogens due to their resistance to last resort antibiotics [2–4], high morbidity and mortality, and the high potential for their resistance to spread via mobile genetic elements [5, 6].

CRE are often associated with healthcare transmission, as demonstrated in the United States, where more than 9000 healthcare-associated infections (HAI) are caused by CRE each year [7, 8]. Healthcare-related risk factors associated with CRE infection include prolonged hospital stay, presence of invasive medical devices, admission to an intensive care unit (ICU), and previous exposure to antimicrobials [9–12]. Data on these risk factors are useful to guide CRE prevention and control efforts, but most data describing CRE epidemiology are reported from high resource settings [13].

The healthcare system in Egypt includes a network of secondary and tertiary healthcare facilities in the public, university, or private sector in 27 geographic regions or governorates. Egypt is one of the first countries in World Health Organization’s (WHO) Eastern Mediterranean Region (EMR) to develop a prospective, standardized national HAI surveillance system. Established in May 2011, Egypt’s HAI surveillance system aims to estimate HAI prevalence and incidence, establish national benchmarks, and describe HAI-causing pathogens in order to inform prevention activities [14, 15]. Using data from Egypt’s national HAI surveillance program, we described HAIs caused by CRE to examine burden, trends, and risk factors associated with CRE HAIs in ICU patients compared to those with carbapenem-susceptible Enterobacteriaceae (CSE) HAIs.

## Methods

### Egyptian HAI surveillance system

Egypt’s HAI surveillance system was implemented in a phased approach, with 310 ICUs in 72 hospitals across 25 governorates collecting data between 2011 to 2017. Hospitals were selected to participate in the surveillance system based on the presence of hospital management support, well-trained infection prevention and control (IPC) teams including link IPC nurses in ICUs, adequate microbiology laboratory capacity (i.e., ability to conduct pathogen identification and antibiotic susceptibility testing [AST]), and data entry capacity. The surveillance system focuses on the four most common types of HAIs in ICU patients as identified by data from the first year of Egypt’s HAI surveillance program [14, 15]: bloodstream infections (BSI), urinary tract infections (UTI), pneumonia (PNA), and surgical site infections (SSI). The methodology of the national HAI surveillance program, including HAI definitions, have been described in earlier publications [14, 15].

### Definitions

HAI surveillance definitions used for BSI, UTI, PNA, and SSI were derived from the Centers for Disease Control and Prevention’s (CDC) 2012 National Healthcare Safety Network (NHSN) [16], with minor adaptations for the primary BSI case definition. Cases of HAI with a culture growing an Enterobacteriaceae from a blood, urine, respiratory tract, or surgical site were included in this analysis. CRE cases were HAIs with Enterobacteriaceae isolates resistant to at least one of the following carbapenems: imipenem, meropenem, ertapenem [17]. CSE cases were HAIs with Enterobacteriaceae isolates not resistant to any of these carbapenems. This analysis was focused on the most common Enterobacteriaceae pathogens according to existing AMR data from Egypt, and included *Klebsiella spp (only K. pneumoniae* and *K. oxytoca* aggregated, but not other rare species), *Escherichia coli* and *Enterobacter spp* [18].

### Microbiological testing

On a monthly basis, all isolates associated with signs or symptoms of infection were sent to the AMR reference laboratory for confirmatory identification and AST for quality control purposes. Antimicrobial susceptibility testing was performed using disk diffusion (Becton Dickinson, USA) according to Clinical and Laboratory Standards Institute guidelines (CLSI) [19]. Only microbiologic confirmatory testing data from the AMR reference lab were used for this analysis to ensure accurate laboratory results.

### Electronic data collection, entry, and reporting

Egypt’s HAI surveillance program uses electronic data collection methods and automated analysis, thereby reducing the workload on hospital HAI surveillance coordinators (SC) and ensuring timeliness of reporting and feedback. To identify cases, the hospital SC screens ICU patients for new clinical signs and symptoms suggestive of infection (fever, crackles, cough, etc.) at least 3 days per week by reviewing medical records, interviewing physicians, and reviewing diagnostic test results (microbiology results or radiology reports). When microbiology results are not available, the physician requests appropriate specimens to be collected for microbiology testing. When a patient is suspected of a HAI based on signs, symptoms, or diagnostic test results, patient information is entered into a standardized case report form installed on the electronic device. The device automatically analyzes the entered data to determine whether the patient meets the HAI case definition and if so, specifies the type of infection. Denominator data (i.e., patient-days, central line–days, urinary catheter–days, ventilator-days) are entered daily on a standardized denominator reporting form installed on the device. The clinical and epidemiological HAI data are uploaded weekly to a secured web-based surveillance application [15] . HAI microbiological data produced by the AMR reference laboratory are also uploaded to the web application, where the data are merged with the HAI clinical and epidemiological data. The web application has built in data quality checks and analytic tools for immediate data cleaning and analysis, which allows hospital teams to generate automated individualized facility reports.

### Data variables

The following patient data were available for each case: patient demographics (i.e., age, sex), hospital and ICU type as defined by NHSN [20, 21], admission and discharge date, length of ICU stay prior to specimen collection, location prior to ICU admission, symptoms that met the case definition, presence of invasive devices, associated surgical procedures in last 90 days, radiological results, and patient outcome (i.e. death, discharged). Relevant microbiology data were also collected including specimen type, specimen collection date, pathogen, and AST results.

### Statistical analysis

Data on CRE and CSE cases identified between May 2011 and December 2017 were included in this analysis. An isolate-level analysis of all Enterobacteriaceae isolates from CRE and CSE cases, including multiple isolates from an individual case, was conducted to calculate the proportion of CRE isolates among all Enterobacteriaceae isolates, stratified by pathogen. A hospital-level analysis was conducted by calculating the proportion of hospitals with ≥1 CRE positive specimen among hospitals conducting HAI surveillance, stratifying by hospital type and size.

For the patient-level analysis, patients were counted as being a CRE case if they had any sample positive for CRE; however, patients with multiple Enterobacteriaceae isolates from the same admission, but from different specimen sites or different dates were counted only once. For patients with multiple isolates, only the first CRE isolate was included in the analysis. Frequencies of categorical variables were stratified by CRE status and compared between CRE and CSE cases using χ2 test or 2-tailed Fisher Exact test. For continuous variables, means with standard deviations (or medians and interquartile ranges if distributions were skewed) were stratified by CRE status and compared between CRE and CSE cases using Student t test or Mann–Whitney test, dependent on the validity of normality assumption.

Univariate analysis was performed using logistic regression to calculate odds ratios, 95% confidence intervals (CI), and *p*-values to determine the strength of the association between these variables and CRE status. The reference category was assigned based on the category with the highest frequency of Enterobacteriaceae HAI cases, except in the analysis of ICU category, where Surgical Critical Care was used as the reference because the neonatal intensive care unit (NICU) was considered too specialized to serve as the reference. Variables with chi-square test *p*-value < 0.10 in the univariate analysis were then included into a multivariable logistic regression model, using a forward stepwise approach to identify risk factors associated with CRE status. Variables were kept in the final model if the p-value for the likelihood ratio test was < 0.05. All analysis were performed using STATA 13 [22].

Incidence rates for CRE cases were calculated as the proportion of CRE cases per 10,000 patient days [20]. For the trend analysis**,** χ2 test was used to determine statistical significance of the trend in CRE case incidence and proportion of CRE cases among all Enterobacteriaceae cases.

## Results

A total of 3836 Enterobacteriaceae isolates from 3109 patients were reported to Egypt’s HAI surveillance system from 2011 to 2017. For the isolate-level analysis, 1105 (47.9%) of the 2306 Enterobacteriaceae isolates submitted to the AMR reference laboratory were CRE [Table 1]. When stratified by pathogen, approximately half of *Klebsiella* (*n* = 929, 53.7%) and *Enterobacter* (*n* = 54, 43.5%) isolates were CR, while a smaller percentage of *Escherichia coli* isolates (*n* = 122, 27.1%) were CRE. The overall incidence of HAI due to CRE was 3.7 per 10,000 patient-days. Among the 72 hospitals performing HAI surveillance, 46 (63.9%) reported at least one CRE isolate during the study period, but this percentage varied by hospital type and hospital size (Table 2).

**Table 1:** *Enterobacteriaceae* isolates* with Carbapenem resistance by type of organism, May 2011 – December 2017

	No. isolates tested for Carbapenem resistance	No. isolates with Carbapenem resistance	% Carbapenem resistant ^*^
*Klebsiella*	1731	929	53.7
*Escherichia coli*	451	122	27.1
*Enterobacter*	124	54	43.5
Total	2306	1105	47.9

*out of all Enterobacteriaceae isolates with all isolates from each patient included

**Table 2:** Number and percentage of hospitals reporting Carbapenem-Resistant Enterobacteriaceae from HAI surveillance by selected characteristics, May 2011 – December 2017

Characteristics	Total no. of hospitals performing HAI surveillance *N* = 72	No. hospitals reporting ≥ 1 CRE positive specimen *N* = 46	% reporting ≥ 1 CRE positive specimen
Hospital type*			
Medical	15	8	53.3
General	30	21	70
Surgical	15	8	53.3
Obstetrics	5	4	80
Pediatrics	7	5	71.4
Hospital size (no. of beds)			
≥ 501	13	8	61.5
201–500	33	18	54.5
≤ 200	26	20	76.9
Total	72	46	63.9

*criteria according to NHSN definitions

For the patient-level analysis, there were 871 CRE cases and 727 CSE cases (Table 3). Blood was the most common specimen type for both CRE cases (47.0%) and CSE cases (33.8%), but CRE was more likely among blood specimens compared to other specimen types (respiratory: OR = 0.65, 95% CI = 0.5–0.85; urine: OR = 0.59, 95% CI = 0.44–0.8; tissue/wound: OR = 0.51, 95% CI = 0.39–0.66). The most common pathogen for CRE cases was *Klebsiella* (85.1%), followed by *E. coli* (10.2%) and *Enterobacter* 4.7%. The median age of CRE cases was 19 years compared to that of CSE cases at 37 years (OR = 0.87, 95% CI = 0.79–0.97).

Most CRE cases were reported from general (*n* = 248, 28.5%) or obstetrical hospitals (*n* = 234, 26.9%). The univariate analysis showed that carbapenem resistance was more common among cases admitted to an obstetrical hospital (OR = 1.36, 95% CI = 1.12–1.82), a smaller sized hospital with ≤200 beds (OR = 2.05, 95% CI = 1.57–2.68), a medical/surgical critical care (CC) unit (OR = 1.59, 95% CI = 1.09–2.31), a NICU (OR = 2.24, 95% CI = 1.66–3.01), and a pediatric cardiothoracic CC unit (OR = 5.77, 95% CI = 1.66–20.05) [Table 3]. The majority of both CRE cases (75.9%) and CSE cases (70.0%) were hospitalized immediately prior to ICU admission. However, CRE cases were more likely to be hospitalized prior to ICU admission (OR = 1.35, 95% CI = 1.1–1.68) and have a longer ICU length of stay prior to infection (OR = 1.31, 95% CI = 1.1–2.1).

**Table 3:** Analysis of risk factors associated with CR and Non-CR cases

Characteristics	Total Enterobacteriaceae HAI cases(*N* = 1598) *n* %		CRE cases (*N* = 871) n %		CSE cases (*N* = 727) n %		Unadjusted OR (95% CI)	*p-value*
Specimen Type								< 0.001a
Blood	655	41.0	409	47.0	246	33.8	Reference	
Respiratory*	346	21.7	180	20.7	166	22.8	0.65 (0.5–0.85)	0.001^b^
Urine	236	14.8	117	13.4	119	16.4	0.59 (0.44–0.8)	0.001 ^b^
Tissue or Wound	361	22.6	165	18.9	196	27.0	0.51 (0.39–0.66)	0.001 ^b^
Type of Enterobacteriaceae Pathogen								< 0.001 ^a^
Klebsiella	1177	73.7	741	85.1	436	60.0	Reference	
Enterobacter	78	4.9	41	4.7	37	5.1	0.65 (0.41–1.03)	0.068 ^b^
E-coli	343	21.5	89	10.2	254	34.9	0.21 (0.16–0.27)	< 0.00 ^b^
Age, years, median (IQR)	28 (1–58)		19 (1–55)		37 (1–61)		0.87 (0.79–0.97)	0.007 ^a^
Patient Sex								0.4 ^a^
Male	859	53.7	475	54.5	384	52.8	Reference	
Female	739	46.3	396	45.5	343	47.2	1.07 (0.88–1.31)	
Hospital Type								< 0.001 ^a^
General	433	27.1	248	28.5	185	25.5	Reference	
Medical	242	15.1	109	12.5	133	18.3	0.61 (0.45–0.84)	0.002 ^b^
Obstetrics	362	22.7	234	26.9	128	17.6	1.36 (1.12–1.82)	0.03 ^b^
Pediatrics	208	13.0	126	14.5	82	11.3	1.15 (0.82–1.61)	0.42 ^b^
Surgical	353	22.1	154	17.7	199	27.4	0.58 (0.43–0.77)	0.001 ^b^
Hospital Size								< 0.001 ^a^
> =501	630	39.4	317	36.4	313	43.1	Reference	
201–500	602	37.7	307	35.3	295	40.6	1.03 (0.82–1.28)	0.81 ^b^
< =200	366	22.9	247	28.4	119	16.4	2.05 (1.57–2.68)	0.001 ^b^
ICU Category								< 0.001 ^a^
Surgical CC	335	21.0	166	19.1	169	23.3	Reference	
Burn CC	54	3.4	13	1.5	41	5.6	0.32 (0.17–0.62)	0.001 ^b^
Medical CC	50	3.1	26	3.0	24	3.3	1.1 (0.61–2)	0.75 ^b^
Medical CC	160	10.0	69	7.9	91	12.5	0.77 (0.53–1.13)	0.18 ^b^
Medical Neurological Care	26	1.6	9	1.0	17	2.3	0.54 (0.23–1.24)	0.15 ^b^
Medical/Surgical CC	169	10.6	103	11.8	66	9.1	1.59 (1.09–2.31)	0.01 ^b^
NICU	419	26.2	288	33.1	131	18.0	2.24 (1.66–3.01)	0.001 ^b^
Neurosurgical Critical Care	101	6.3	48	5.5	53	7.3	0.92 (0.59–1.44)	0.72 ^b^
Ped. Medical Critical Care	77	4.8	40	4.6	37	5.1	1.1 (0.67–1.81)	0.71 ^b^
Pediatric Cardiothoracic CC	20	1.3	17	2.0	3	0.4	5.77 (1.66–20.05)	0.006 ^b^
Prenatal/Surgical	33	2.1	15	1.7	18	2.5	0.85 (0.41–1.74)	0.65 ^b^
Respiratory CC	44	2.8	25	2.9	19	2.6	1.34 (0.71–2.52)	0.36 ^b^
Surgical Cardiothoracic CC	29	1.8	8	0.9	21	2.9	0.39 (0.17–0.9)	0.02 ^b^
Trauma CC	81	5.1	44	5.1	37	5.1	1.21 (0.74–1.97)	0.44 ^b^
Hospitalized prior to ICU admission								0.008 ^a^
No	428	26.8	210	24.1	218	30.0	Reference	
Yes	1.170	73.2	661	75.9	509	70.0	1.35 (1.1–1.68)	
Surgical Procedure during hospital admission								< 0.001 ^a^
No	972	60.8	561	64.4	411	56.5	Reference	
Yes	626	39.2	310	35.6	316	43.5	0.72 (0.59–0.88)	
Mechanical ventilation								
No	713	44.6	377	43.3	336	46.2	Reference	0.08^a^
Yes	885	55.4	494	56.7	391	53.8	1.13 (0.92–1.37)	
Urinary Catheter								< 0.001 ^a^
No	579	36.2	374	42.9	205	28.2	Reference	
Yes	1.019	63.8	497	57.1	522	71.8	0.52 (0.42–0.64)	
Central Line								0.89 ^a^
No	537	33.6	294	33.7	243	33.4	Reference	
Yes	1.061	66.4	577	66.3	484	66.6	0.99 (0.8–1.21)	
LOS in ICU prior to specimen collection, median, days, (IQR)	7 (3–13)		8 (3–15)		5 (2–11)		1.31 (1.1–2.1)	< 0.001 ^a^
Patient outcome								< 0.001 ^a^
Discharged/ Transferred	690	43.2	339	38.9	351	48.3	Reference	
Died	908	56.8	532	61.1	376	51.7	1.46 (1.2–1.79)	

*a p-value generated using chi-square test or 2-tailed Fisher Exact test for categorical variables and Student t test or Mann–Whitney test, dependent on the validity of normality assumption for continuous variables*

*b p-values generated using the logistic regression for specific categories of non-dichotomous variables*

*c Include:* Deep Tracheal Aspirate & *BAL: Broncho alveolar lavage*

*d includes both hospital where Enterobacteriaceae HAI occurred and other hospitals*

CRE cases were less likely to have underwent a surgical procedure (OR = 0.72, 95% CI = 0.59–0.88) or had a urinary catheter during the hospital admission (OR = 0.52, 95% CI = 0.42–0.64), but there was no significant difference between CRE and CSE cases with respect to mechanical ventilation or central lines. Mortality was also higher among CRE cases than CSE cases (OR = 1.46, 95% CI = 1.2–1.79). When adjusted for patient and specimen characteristics, the multi-variate analysis showed that hospitalization immediately prior to ICU admission (OR = 1.38; 95% CI: 1.18–1.76; *p*-value 0.008) and a longer ICU stay prior to specimen collection (OR = 1.08; 95% CI: 1.14–1.36; *p*-value 0.03) remained significantly associated with carbapenem resistance, while infection with *E. coli* (OR = 0.22; 95% CI: 0.17–0.3; p-value < 0.001) and identification in a wound specimen (OR = 0.67; 95%CI: 0.5–0.89; p-value 0.01) were associated with not having carbapenem resistance. The association between carbapenem resistance and specific hospital or ICU types did not remain significant in the multivariable analysis.

**Fig. 1:**
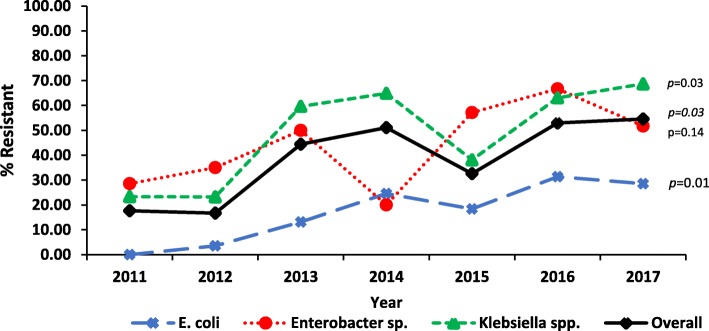
Proportion of Enterobacteriaceae isolates with Carbapenem resistance by year 2011–2017

**Fig. 2:**
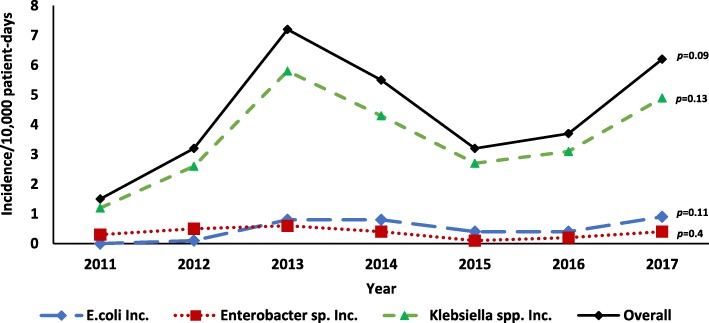
Incidence of Carbapenem Resistant Enterobacteriaceae (CRE) infections per 10,000 patient-days by year 2011–2017

There was an overall increase in the proportion of cases that were CRE (17.6 to 54.6%, *p* = 0.003), which remained when stratified by pathogen (Fig. 1). Although not statistically significant, the incidence of carbapenem resistance cases for all pathogens increased between 2011 and 2013, followed by a decline between 2013 and 2015. Since 2015, the incidence overall and for each pathogen has again been increasing (Fig. 2).

## Discussion

Our analysis found that carbapenem resistance is widespread and the prevalence is increasing in Egypt. We found that more than half of hospitals (64%) had at least one CRE isolate and half (47.9%) of Enterobacteriaceae isolates were CRE, which is higher than estimates reported from other Arab, African, or Asian countries [23–26]. The incidence of CRE HAI (3.7/10,000 patient-days) is also much higher than the overall incidence of all CRE (HAI and non-HAI) reported from other countries, including the United States (0.1–0.4/10,000 patient-days) Canada (0.2 per 10,000 patient-days), and China (0.4 per 10,000 patient-days) [27–29].

The severity of the CRE problem in Egypt emphasizes the importance of healthcare facilities’ to implement CRE prevention and control efforts. The Centers for Disease Control and Prevention (CDC) and the WHO have developed guidance for healthcare facilities on strategies proven to be effective for limiting CRE transmission, including IPC, antimicrobial stewardship, and CRE surveillance [29, 30]. Data from this analysis can be used to guide how and where these strategies can be most efficiently implemented in Egypt.

Potential causes of the high prevalence of CREs in several hospitals in Egypt might be due to the limitation in implementing stewardship programs and IPC measures. IPC activities are a critical part of preventing healthcare-related CRE transmission and include practices such as hand hygiene, minimizing device use, environmental cleaning, and isolation through contact precautions and improve cohorting of patients or staff. Multivariate analysis did not identify specific types of hospitals or ICUs with higher likelihood of carbapenem resistance, which would help target IPC interventions. Rapid identification and reporting of CRE in clinical labs may help to demonstrate where to target prevention efforts in the future.

Previous global studies have found that CRE is associated with increased length of ICU stay, undergoing surgical procedures, and use of medical devices, specifically mechanical ventilation and central venous catheters [9, 33–35]. Our multivariable analysis did not find that carbapenem resistance was significantly associated with exposure to medical devices or surgical procedures. This may be explained by the very high frequency of invasive devices used in Egyptian ICUs, among both CRE and CSE cases. However, this finding should not discount the importance of CRE prevention strategies focused on reducing healthcare exposures, such as device utilization.

We identified a relative decrease in CRE incidence and proportion of carbapenem resistance in 2014–2015. The introduction of an analytic tool in 2013 may have contributed to the reduction in CRE incidence by providing more timely data feedback to hospitals for guiding IPC interventions. The high frequency of CR found in blood and the increased odds of carbapenem resistance among blood may reflect culturing practices, where blood cultures may only be ordered for very ill patients with resistant infections refractory to treatment.

The ability to adequately contain CRE requires sufficient microbiology laboratory testing to ensure accurate CRE detection and timely notification of laboratory results. In this surveillance system, some of the best laboratories in the country are included; the burden and epidemiology in other facilities, particularly those without reliable susceptibility testing, is largely unknown. Although global guidance recommends active surveillance by conducting CRE screening of patients in some situations with an outbreak or ongoing high prevalence of CRE, this practice is not routine in Egypt, and further efforts to implement such activities are likely needed.

The limited number of studies published about CRE in Egypt have involved few facilities and focused mainly on identifying genetic resistance mechanisms, rather than epidemiological risk factors [36–38]. This study uses data from many healthcare facilities across Egypt, thereby strengthening evidence about epidemiological risk factors associated with CRE. While there are different types and sizes of healthcare facilities included, they are a convenience sample and do not constitute a representative sample of the country.

Detection of CRE in this network was also limited by inconsistent microbiology and surveillance capacity at hospital laboratories. Some isolates were not tested for carbapenem susceptibility at the hospital laboratory or were not sent to the AMR reference laboratory for confirmatory testing, resulting in data for these samples not being included in the data analysis. Because active screening for CRE was not being performed, the variable propensity for clinicians to test patients also likely impacted CRE detection. The trend analysis does not take into account changes at the national, facility, or ward level which impact CRE detection, including changes in specimen collection and testing.

The scope of this surveillance system is limited to ICU locations since ICUs are expected to have both the highest risk for transmission and the most vulnerable population. Therefore, this data cannot be used to draw any conclusions about CRE burden or risk factors in non-ICU wards or the community. This surveillance system did not collect data on clinical variables which prior studies have found to be associated with CRE infections, such as patients’ comorbid medical conditions or prior antibiotic exposure.

## Conclusions

This study shows that CRE is prevalent and increasing in Egyptian hospitals, suggesting the presence of selective pressures and healthcare transmission. Future implementation of evidence-based IPC strategies to prevent CRE transmission, strengthening microbiology capacity and molecular characterization in addition to including antibiotic stewardship programs, are needed to reduce the burden of CRE and optimize patient treatment strategies. CRE surveillance should be strengthened to better track the incidence and prevalence of this pathogen and define the impact of interventions on the burden of this serious, emerging threat.

## Data Availability

The datasets used and/or analysed during the current study are available from the corresponding author on reasonable request.

## Abbreviations

AMR: Antimicrobial resistance
AST: Antibiotic susceptibility testing
BSI: Bloodstream infections
CDC: Centers for Disease Control and Prevention
CI: Confidence intervals
CRE: Carbapenem-resistant Enterobacteriaceae
CSE: Carbapenem- susceptible Enterobacteriaceae
EMR: Eastern Mediterranean Region
HAI: Healthcare-associated infections
ICU: Intensive care unit
NHSN: National Healthcare Safety Network
NICU: Neonatal intensive care unit
PNA: Pneumonia
SC: Surveillance coordinators
SSI: Surgical site infections
USAID: U.S. Agency for International Development
UTI: Urinary tract infections
WHO: World Health Organization’s
